# Supplementary material for: Real‐time cardiovascular MR with spatio‐temporal artifact suppression using deep learning–proof of concept in congenital heart disease
Source: Magn Reson Med. 2018 Sep 8;81(2):1143–56. doi: 10.1002/mrm.27480 (PMC6492123; doi:10.1002/mrm.27480)
Supplement: Supplementary file 1 — FIGURE S1 Flow diagram showing the steps taken to convert the retrospectively cardiac‐gated, BH‐bSSFP data to synthetic real‐time data used to train the residual U‐Net. FIGURE S2 Effect of number of iterations on GRASP reconstruction speed and GRASP reconstruction accuracy. Preliminary experiments showing (A) average reconstruction time per slice for different number of iterations (red line shows average time per slice for 50 iterations, as used in this study), (B) resultant image quality in 1 patient for different numbers of iterations, (C) plot of difference in resultant images between different numbers of iterations (red line shows difference in images between 50‐60 iterations), and (D) difference images between numbers of iterations. FIGURE S3 Assessment of robustness: effect of SNR. (A) Average RMSE and SSIM results across all 222 test data sets at different levels of SNR. (B) Plot of RMSE at different SNR levels. (C) Plot of SSIM at different SNR levels. (B and C) Red line shows reference RMSE/SSIM with no additional noise. (D) Example of image quality from 1 patient with input data from different SNR levels. FIGURE S4 Assessment of robustness: effect of acceleration. (A) Average RMSE and SSIM results across all 222 test data sets at different acceleration factors. (B) Plot of RMSE at different acceleration factors. (C) Plot of SSIM at different acceleration factors. (B and C) Red line shows reference RMSE/SSIM at 13× (this is the acceleration factor the network was trained with). (D) Example of image quality from 1 patient. FIGURE S5 Assessment of robustness: effect of shifting the cropping region. (A) Average RMSE at different cropping positions in x and y across all 222 test data sets. (B) Plot of RMSE at different a cropping positions. (C) Average SSIM at different cropping positions in x and y across all 222 test data sets. (D) Plot of SSIM at different cropping positions. (E) Example of image quality from 1 patient. FIGURE S6 Bland‐Altman plot comparing [file MRM-81-1143-s001.docx]

Supporting Information Text S1

*Diagnosis of patients used for training of the residual U-Net, creating of the synthetic test data, and for the prospective study.*

**Training data (N=250)**

Repaired tetralogy of Fallot/pulmonary atresia with ventricular septal defect; 140

Normal; 38 (Family history cardiomyopathy)

Cardiomyopathy; 31

Coarctation of the aorta; 21

Aortic valve disease; 15

Repaired transposition of the great arteries; 4

Atrial septal defect; 1

**Synthetic Test data (N=25)**

Repaired tetralogy of Fallot/pulmonary atresia with ventricular septal defect; 16

Aortic valve disease; 2

Coarctation of the aorta; 3

Cardiomyopathy; 2

Repaired transposition of the great arteries; 2

**Prospective real-time data (N=10)**

Repaired tetralogy of Fallot/pulmonary atresia with ventricular septal defect; 4

Coarctation of the aorta; 2

Aortic valve disease; 2

Atrial septal defect; 2

**Supporting Information Figure S1**

**Supporting Information Figure S2**

**Supporting Information Figure S3**

**Supporting Information Figure S4**

**Supporting Information Figure S5**

**Supporting Information Figure S6**

**Supporting Information Figure S7**

**Supporting Information Figure S8**
